# Physiological and Biochemical Responses of *Illicium verum* Seedlings to Drought

**DOI:** 10.3390/ijms27177509

**Published:** 2026-08-22

**Authors:** Miaojuan Guan, Mengwei Wu, Shuai Du, Xiang Luo, Ding Huang, Yong Tan

**Affiliations:** College of Pharmacy, Guangxi University of Traditional Chinese Medicine, Nanning 530200, China; 13647755861@163.com (M.G.); nananzi@163.com (M.W.); dushuai2023@stu.gxtcmu.edu.cn (S.D.); l@gxtcmu.edu.cn (X.L.); hdh016@126.com (D.H.)

**Keywords:** *Illicium verum*, drought stress, osmotic adjustment, antioxidant enzymes, drought resistance evaluation

## Abstract

This study analyzed the changes in physiological and biochemical indexes of seedlings from different *Illicium verum* Hook.f. (*I. verum*) cultivars under drought stress to comprehensively compare their drought resistance and lay a foundation for the screening and artificial cultivation of stress-resistant *I. verum* cultivars. The effects of drought stress on Relative Water Content (RWC), Malondialdehyde (MDA), osmotic adjustment substances, and antioxidant enzymes of seedlings of different *I. verum* cultivars were determined by indoor hydroponic method and PEG-6000 simulated drought stress experiment. RWC showed a downward trend, and the smallest change was Dongrong. The change trend of MDA content was increased and then decreased, and the greatest change was Heiye. Differences exist in the changes of osmotic adjustment substances in seedlings of different *I. verum* cultivars in response to drought stress. The contents of osmoregulatory substances all showed an upward trend. The Proline (Pro) content and soluble sugar (Ss) content in Luoma changed the most, and the soluble protein (Sp) content changed the most in Dongrong. Seedlings of different *I. verum* cultivars differ in antioxidant enzyme activities in response to drought stress. With the extension of drought stress time, the activities of Superoxide dismutase (SOD) enzyme, Peroxidase (POD) enzyme, and Catalase (CAT) enzyme increased first and then decreased. The activities of SOD enzyme, POD enzyme, and CAT enzyme changed the most in Luoma, Dongrong, and Chenping. The drought resistance of the five *I. verum* cultivars was Dongrong > Muwang > Luoma > Heiye > Chenping. Different drought-resistant cultivars should be selected according to local conditions in the cultivation process.

## 1. Introduction

*Illicium verum* Hook.f. (*I. verum*) is an important medicinal and edible economic tree species, which is an evergreen tree of Magnoliaceae. The fruit of *I. verum* is a traditional Chinese medicine. It is warm in nature and pungent in taste. It has the effects of warming yang and dispelling cold, regulating qi, and relieving pain [1]. *I. verum* has unique requirements for the growth environment. It is fond of the natural conditions of a warm and humid climate that is foggy and humid all year round. It is suitable for growing in the deep and fertile mountainside and foot of the mountain. As one of the most common and destructive environmental stresses, drought severely limits the growth and yield of medicinal plants [2]. In recent years, due to the increasing frequency of extreme weather, *I. verum* often suffers from drought damage, resulting in a decline in yield and quality.

Under drought stress conditions, plants will produce adaptive regulatory responses to drought stress by changing their own morphological structure, regulating physiological and biochemical processes, and adjusting gene expression levels, thereby maintaining their normal growth and development [3]. Under drought stress, most plants can enhance their anti-dehydration ability and cell water retention ability by adjusting the relative water content, proline content, soluble protein content, and soluble sugar content of leaves, so as to improve their adaptability to drought stress [4]. To cope with drought stress, plant cells rapidly elevate the levels of antioxidant enzymes including SOD, CAT, and POD. These enzymes safeguard the structural and functional integrity of cell membranes and sustain regular cellular physiological metabolism [5]. When severe oxidation injures cell membranes, malondialdehyde (MDA) accumulates, and its concentration indicates the extent of membrane oxidative injury [6]. Studies have shown that PEG-6000 treatment of plant seedlings can achieve the purpose of dehydration of seedlings, and PEG-6000, as an inorganic polymer compound, does not produce biochemical reactions with plants. It is an ideal experimental method to simulate drought stress of seedlings [7].

To date, researchers have explored the molecular basis of *I. verum* [8]. Additional investigations have focused on its cultivation practices, phytochemical profiles, and pharmacological effects [9]. However, the response mechanism of *I. verum* to drought stress at the physiological level is not yet clear. Therefore, this study adopted PEG-6000 to simulate drought stress, measured physiological indices in leaves of five *I. verum* seedling cultivars, and elucidated the physiological and biochemical regulatory mechanisms underlying drought resistance of *I. verum*. Furthermore, the differences in drought tolerance among seedling cultivars were comprehensively compared, providing theoretical support for seedling propagation, breeding of superior stress-resistant cultivars, and standardized cultivation of *I. verum*.

## 2. Results

### 2.1. Effects of Drought Stress on RWC in Leaves of Five I. verum Cultivars Seedlings

**Figure 1:**
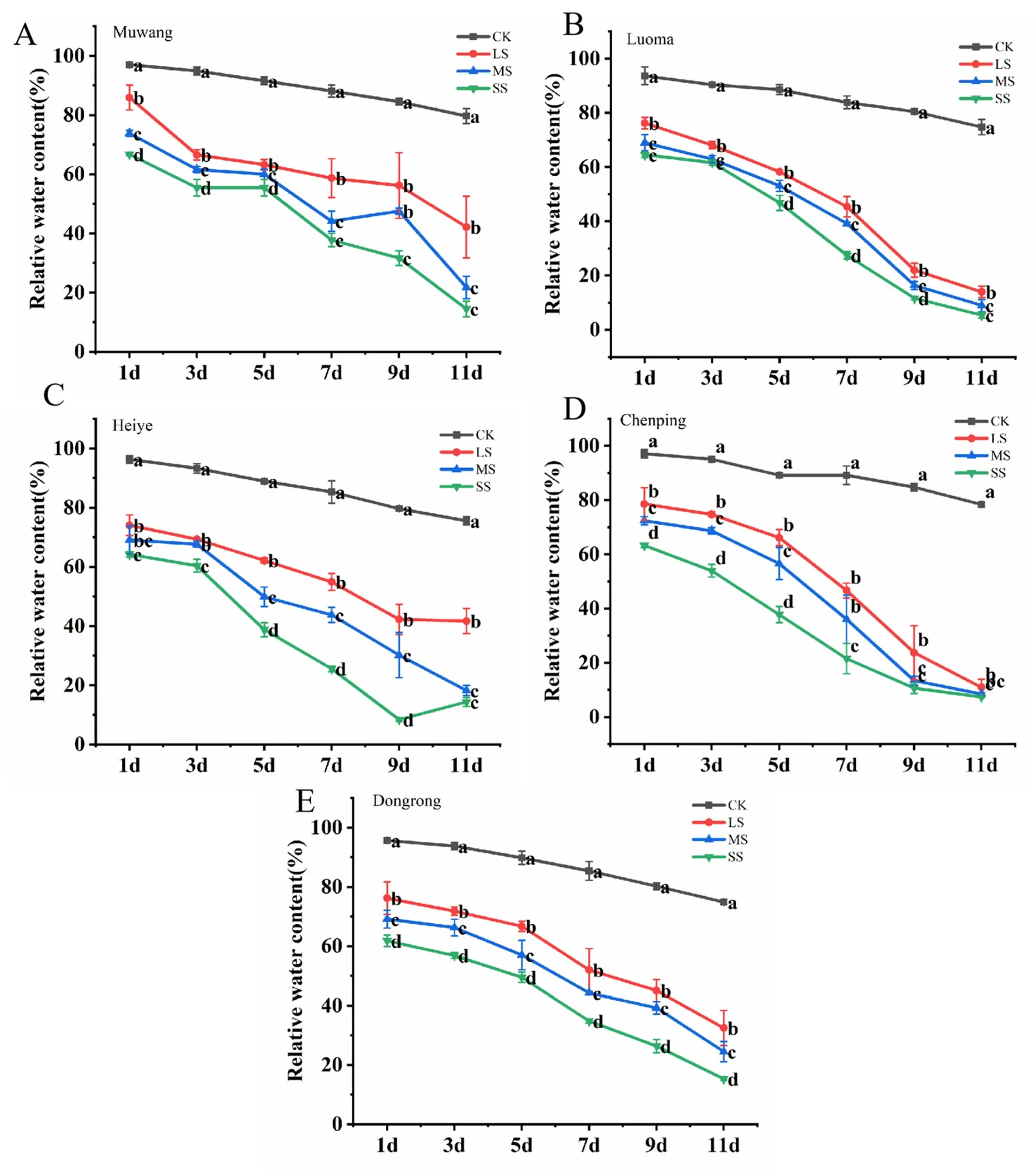
Effects of drought stress on RWC of five *I. verum* cultivars. (**A**)—Muwang; (**B**)—Louma; (**C**)—Heiye; (**D**)—Chenping; (**E**)—Dongrong. Different lowercase letters indicate significant differences between treatments (*p* < 0.05).

Leaf relative water content (RWC) decreased with the intensification of drought stress, and the magnitude of decline varied among cultivars, reaching the minimum value under severe stress. The RWC of control treatments remained at 74–98% throughout the experiment, with an average value higher than that of all drought-stressed groups. On the 11th day of drought stress, under LS, the RWC reduction ranged from 44.84% to 86.08%; Chenping exhibited the largest decrease, while Heiye showed the smallest decline. Under moderate stress MS, the reduction ranged from 67.27% to 89.23%; Chenping had the most obvious drop, and Dongrong had the mildest reduction. Under SS, the reduction ranged from 79.57% to 92.78%; Luoma presented the greatest decrease, whereas Dongrong had the least decline. A large number of leaves of Chenping wilted and shed during stress, which may be a strategy to maintain water balance in the remaining leaves by reducing leaf biomass (Figure 1).

### 2.2. Effects of Drought Stress on MDA Content of Five I. verum Cultivars Seedlings

**Figure 2:**
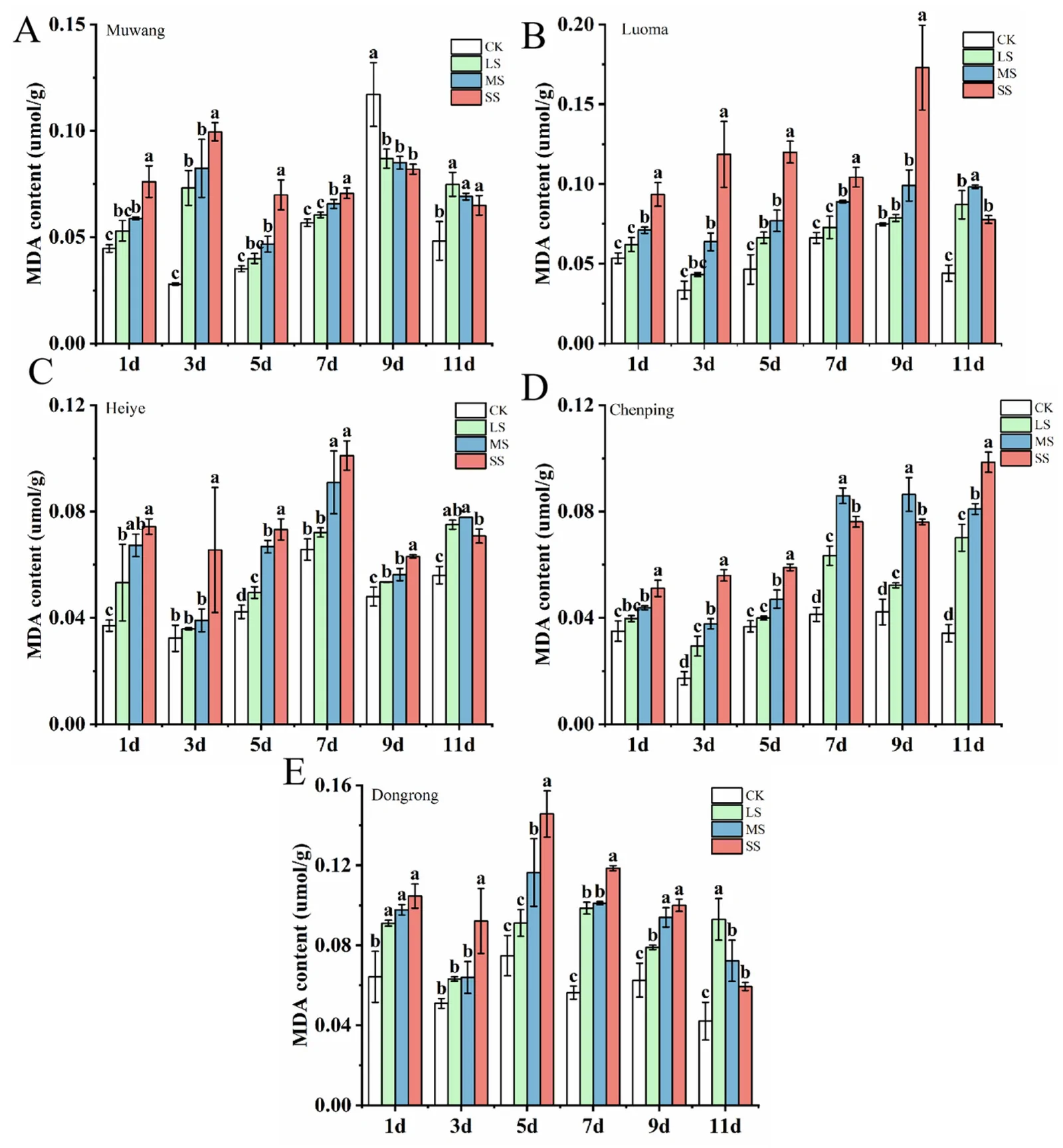
Effects of drought stress on malondialdehyde content of five *I. verum* cultivars. (**A**)—Muwang; (**B**)—Louma; (**C**)—Heiye; (**D**)—Chenping; (**E**)—Dongrong. Different lowercase letters indicate significant differences between treatments (*p* < 0.05).

The malondialdehyde (MDA) content of Muwang showed a trend of rising–falling–rising (Figure 2A); Luoma, Heiye and Chenping displayed the pattern of falling–rising–falling (Figure 2B–D), while Dongrong presented an initial increase followed by a decrease (Figure 2E). On day 11 of drought stress, MDA levels under LS, MS, and SS treatments were 55%, 43% and 35% higher than CK for Muwang; 97%, 123%, and 76% for Luoma; 34%, 39% and 26% for Heiye; 105%, 137% and 188% for Chenping; and 121%, 72% and 41% for Dongrong, respectively.

Among all cultivars, Heiye exhibited the lowest MDA accumulation and the smallest variation range under LS, MS, and SS treatments, suggesting that this cultivar could effectively reduce membrane damage under drought stress and thus possess strong drought resistance. Luoma had the most dramatic MDA variation under light drought, which indicated its cell membranes suffered obvious oxidative damage and it was sensitive to mild water deficit. As stress intensified, its stress-resistant mechanisms were activated, reducing the degree of membrane injury. Under moderate and severe drought, Chenping displayed remarkably higher MDA contents than other cultivars, reflecting severe cell membrane damage and intense membrane lipid peroxidation. These results revealed that Chenping has weak tolerance to moderate or severe drought, with its membrane system suffering the most severe injury under sustained water deficit.

### 2.3. Effects of Drought Stress on Osmotic Adjustment Substances in Seedlings of Five I. verum Cultivars

#### 2.3.1. Effects of Drought Stress on Proline Content in Seedlings of Five *I. verum* Cultivars

**Figure 3:**
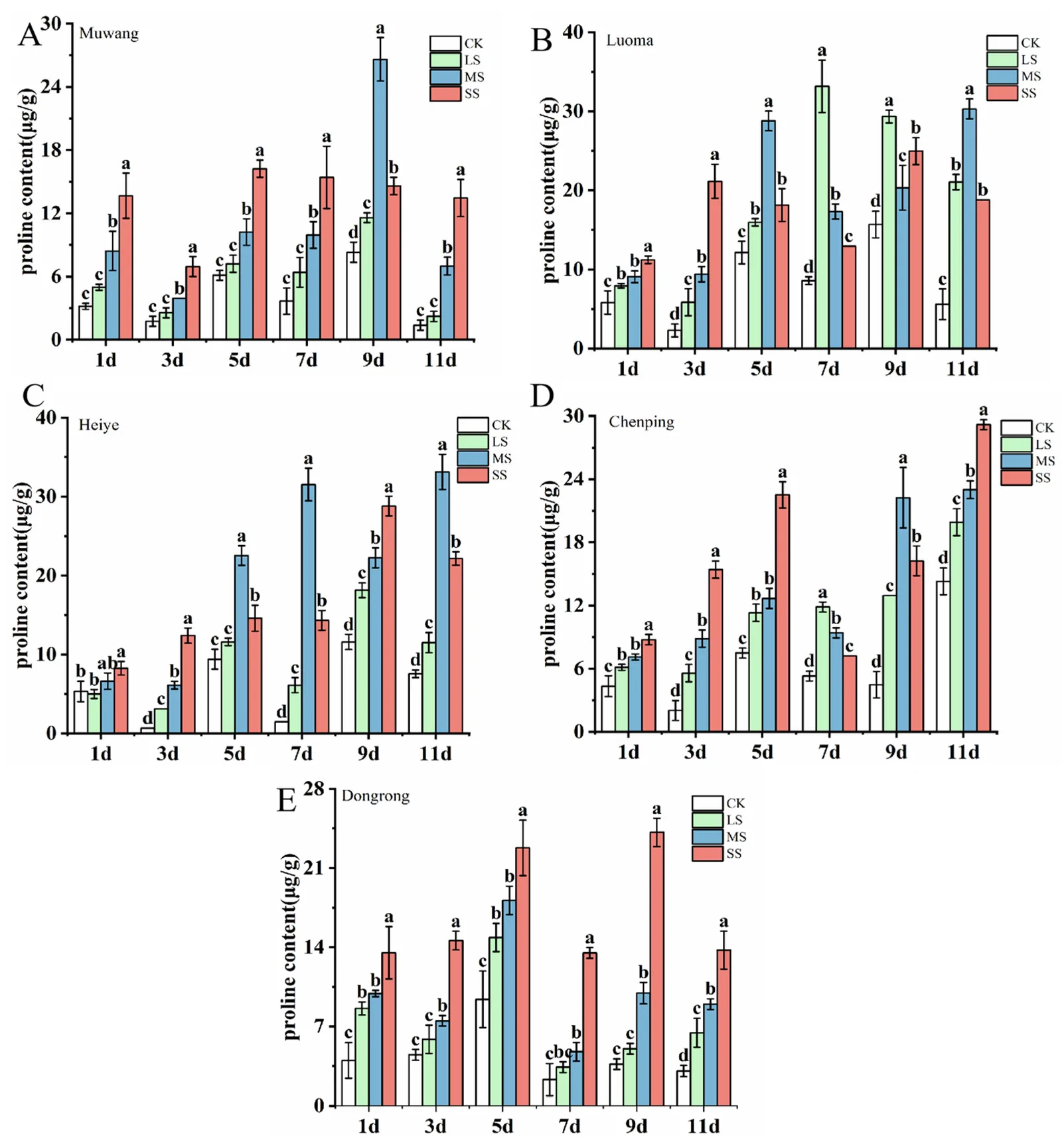
Effects of drought stress on proline content of five *I. verum* cultivars. (**A**)—Muwang; (**B**)—Louma; (**C**)—Heiye; (**D**)—Chenping; (**E**)—Dongrong. Different lowercase letters indicate significant differences between treatments (*p* < 0.05).

The proline (Pro) contents of Muwang, Luoma, Chenping and Dongrong increased initially and then decreased (Figure 3A,B,D,E), whereas the Pro content of Heiye showed an overall upward trend (Figure 3C). On the 11th day of drought stress, the Pro contents of Muwang under LS, MS and SS treatments were 0.61-, 4.08- and 8.78-fold higher than the control (CK), respectively. The corresponding multiples for Luoma were 2.76, 4.42 and 2.36; for Heiye 0.52, 3.38 and 1.93; for Chenping 0.39, 0.61 and 1.04; and for Dongrong 1.10, 1.93 and 3.49.

Luoma exhibited the greatest changes in Pro content under LS and MS treatments, which indicated that this cultivar synthesizes abundant proline to reduce cellular osmotic potential and presents a rapid drought stress response. Muwang accumulated the least proline under light stress, demonstrating weak osmotic adjustment capacity at mild water deficit; nevertheless, it produced the largest amount of proline under severe stress to resist severe dehydration. By contrast, Chenping maintained the slightest Pro elevation under both moderate and severe drought, revealing that it cannot effectively mitigate drought-induced damage via proline accumulation.

#### 2.3.2. Effects of Drought Stress on Soluble Protein Content of Five *I. verum* Cultivar Seedlings

**Figure 4:**
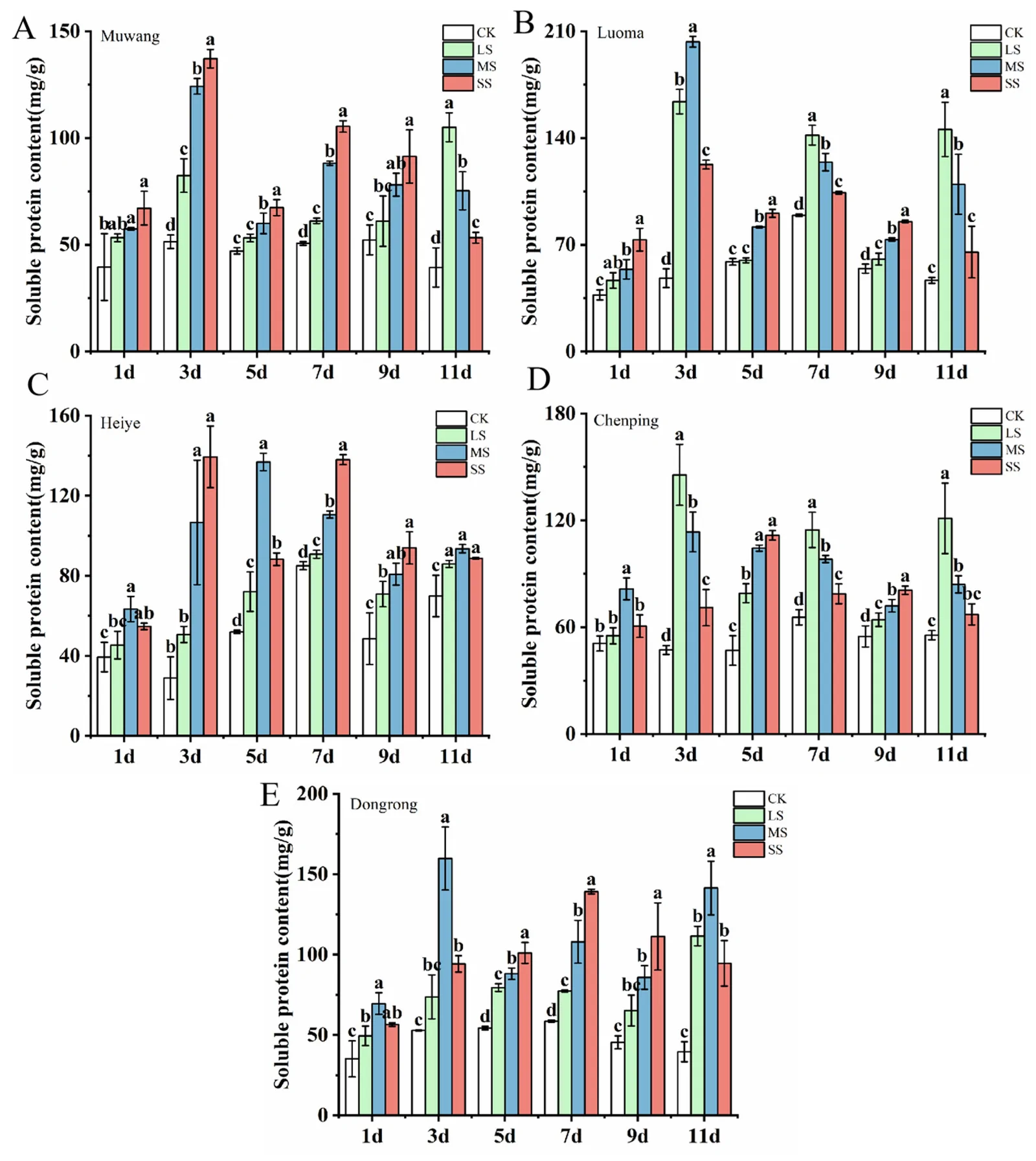
Effects of drought stress on soluble protein content of five *I. verum* cultivars. (**A**)—Muwang; (**B**)—Louma; (**C**)—Heiye; (**D**)—Chenping; (**E**)—Dongrong. Different lowercase letters indicate significant differences between treatments (*p* < 0.05).

Soluble protein (Sp) contents of Muwang and Dongrong showed a rise–fall–rise tendency (Figure 4A,E), while Luoma, Heiye and Chenping exhibited an initial increase followed by a decline (Figure 4B–D). On the 11th day of drought stress, Sp levels of Muwang under LS, MS and SS treatments were 1.67-, 0.91- and 0.35-fold higher than the control (CK), respectively. The corresponding fold increases were 2.12, 1.35 and 0.40 for Luoma; 0.23, 0.34 and 0.27 for Heiye; 1.18, 0.51 and 0.21 for Chenping; and 1.82, 2.57 and 1.39 for Dongrong.

Dongrong displayed the most prominent variations in soluble protein content across LS, MS and SS treatments, which suggested that this cultivar develops an active drought acclimation strategy by accumulating soluble protein. Higher soluble protein can boost cellular osmotic pressure, stabilize macromolecular structures, and improve water retention and dehydration resistance in cells. Heiye had the slightest changes in Sp under LS and MS, indicating limited protein synthesis under light and moderate drought, with mild damage to cell membrane and cellular structures. Chenping showed the minimum fluctuation of Sp under SS treatment, implying relatively weak tolerance to severe drought stress.

#### 2.3.3. Effects of Drought Stress on Soluble Sugar Content of Five *I. verum* Cultivar Seedlings

**Figure 5:**
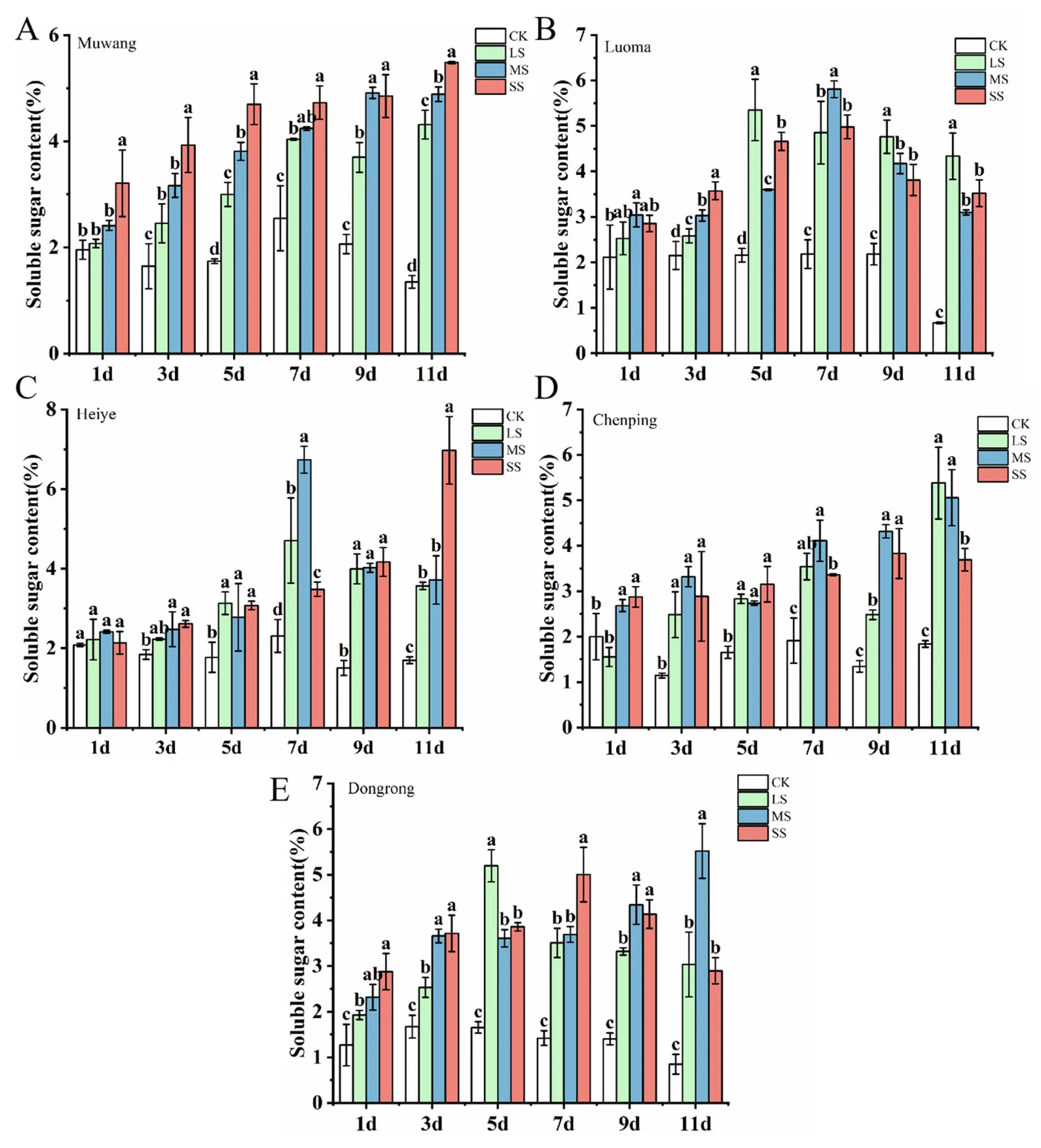
Effects of drought stress on soluble sugar content of five *I. verum* cultivars. (**A**)—Muwang; (**B**)—Louma; (**C**)—Heiye; (**D**)—Chenping; (**E**)—Dongrong. Different lowercase letters indicate significant differences between treatments (*p* < 0.05).

The total soluble sugar contents of Muwang, Heiye and Chenping displayed an overall upward trend (Figure 5A,C,D), whereas those of Luoma and Dongrong first increased and then decreased (Figure 5B,E). On the 11th day of drought stress, soluble sugar contents of Muwang under LS, MS and SS treatments were 2.19-, 3.62- and 4.06-fold higher than CK, respectively. The corresponding fold increases were 5.48, 4.63 and 5.26 for Luoma; 1.10, 2.19 and 4.10 for Heiye; 1.93, 2.75 and 2.01 for Chenping; and 2.58, 6.51 and 3.42 for Dongrong.

Luoma presented the most dramatic changes in soluble sugar under LS and SS treatments, revealing that this cultivar can rapidly trigger the synthesis and accumulation of soluble sugars at the early stress stage and under severe drought. The accumulated sugars elevate cellular osmotic potential to strengthen water retention capacity and alleviate drought-induced damage. Dongrong showed the maximum elevation of soluble sugar under MS, which meant it stabilized cellular structures via massive soluble sugar accumulation.

Heiye had the slightest variations in soluble sugar under LS and MS, with mild damage to its cell membrane system; thus, it did not require massive sugar accumulation to resist drought stress. Chenping exhibited the smallest change in soluble sugar under SS treatment, indicating that it cannot effectively cope with extreme drought through soluble sugar accumulation.

### 2.4. Effects of Drought Stress on Enzyme Activities of I. verum Seedlings

#### 2.4.1. Effects of Drought Stress on SOD Activity of Five *I. verum* Cultivar Seedlings

**Figure 6:**
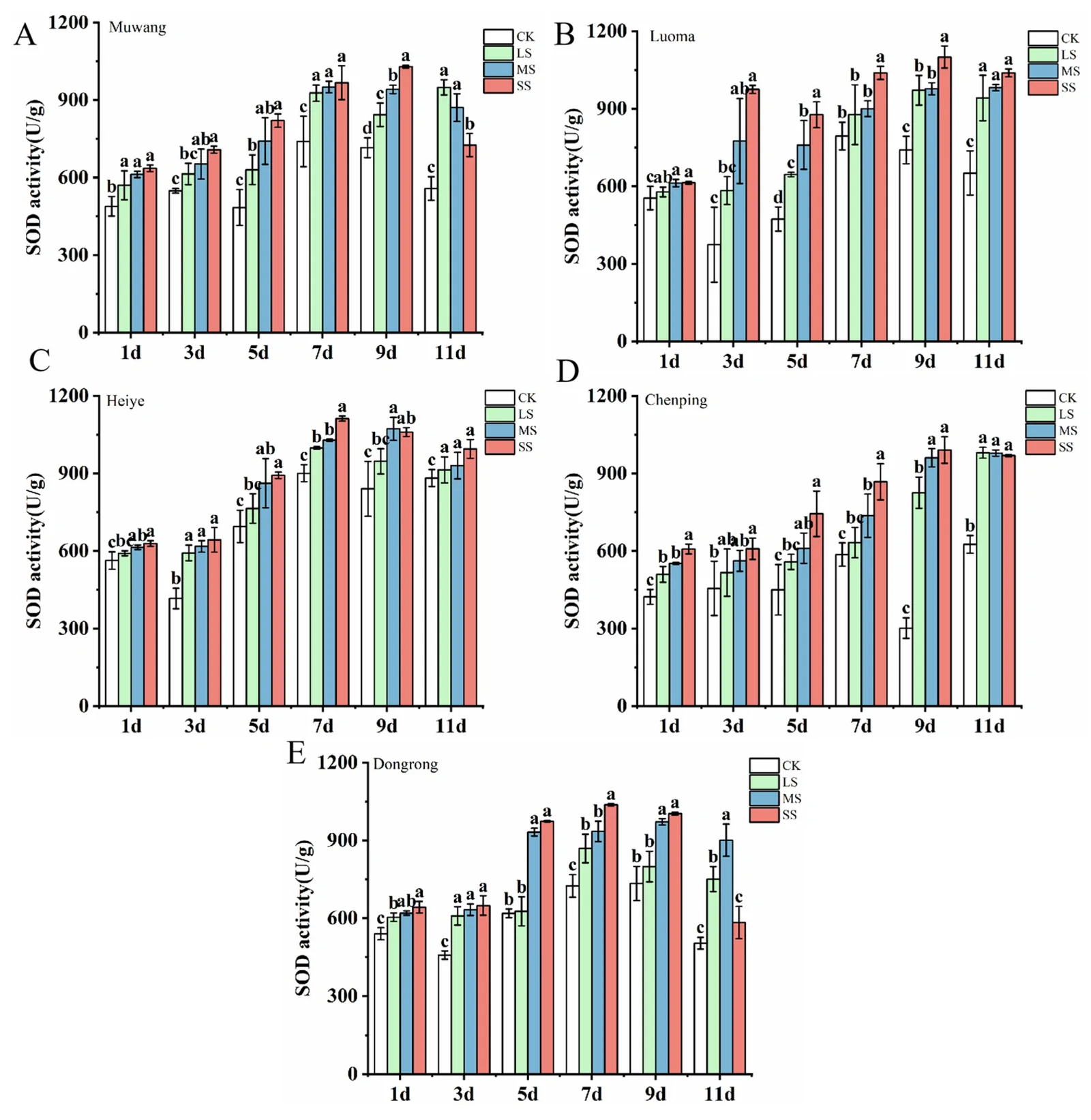
Effects of drought stress on SOD activity of five *I. verum* cultivars. (**A**)—Muwang; (**B**)—Louma; (**C**)—Heiye; (**D**)—Chenping; (**E**)—Dongrong. Different lowercase letters indicate significant differences between treatments (*p* < 0.05).

The superoxide dismutase (SOD) activities of Muwang, Heiye and Dongrong generally rose first and then declined (Figure 6A,C,E), whereas the SOD activities of Luoma and Chenping displayed a gradual upward trend (Figure 6B,D). On the 11th day of drought stress, compared with the control (CK), SOD activities of Muwang under LS, MS, and SS treatments were 0.70-, 0.56- and 0.30-fold higher, respectively. The corresponding fold increases were 0.45, 0.51 and 0.59 for Luoma; 0.04, 0.05, and 0.13 for Heiye; 0.57, 0.56, and 0.55 for Chenping; and 0.16, 0.79 and 0.49 for Dongrong.

Heiye had the slightest variations in SOD activity under LS, MS, and SS treatments, which reflected more stable cell membrane systems and slighter oxidative stress under water deficit. Muwang showed the greatest change in SOD activity under LS, suggesting it is sensitive to early water deficit signals and alleviates oxidative injury by upregulating SOD activity. Dongrong presented the most remarkable elevation of SOD activity under MS, as its whole antioxidant system was fully activated to counteract oxidative damage. Luoma exhibited the largest fluctuation in SOD activity under SS and coped with adverse effects from severe water deficit via dramatic enhancement of SOD activity.

#### 2.4.2. Effects of Drought Stress on POD Activity of Five *I. verum* Cultivar Seedlings

**Figure 7:**
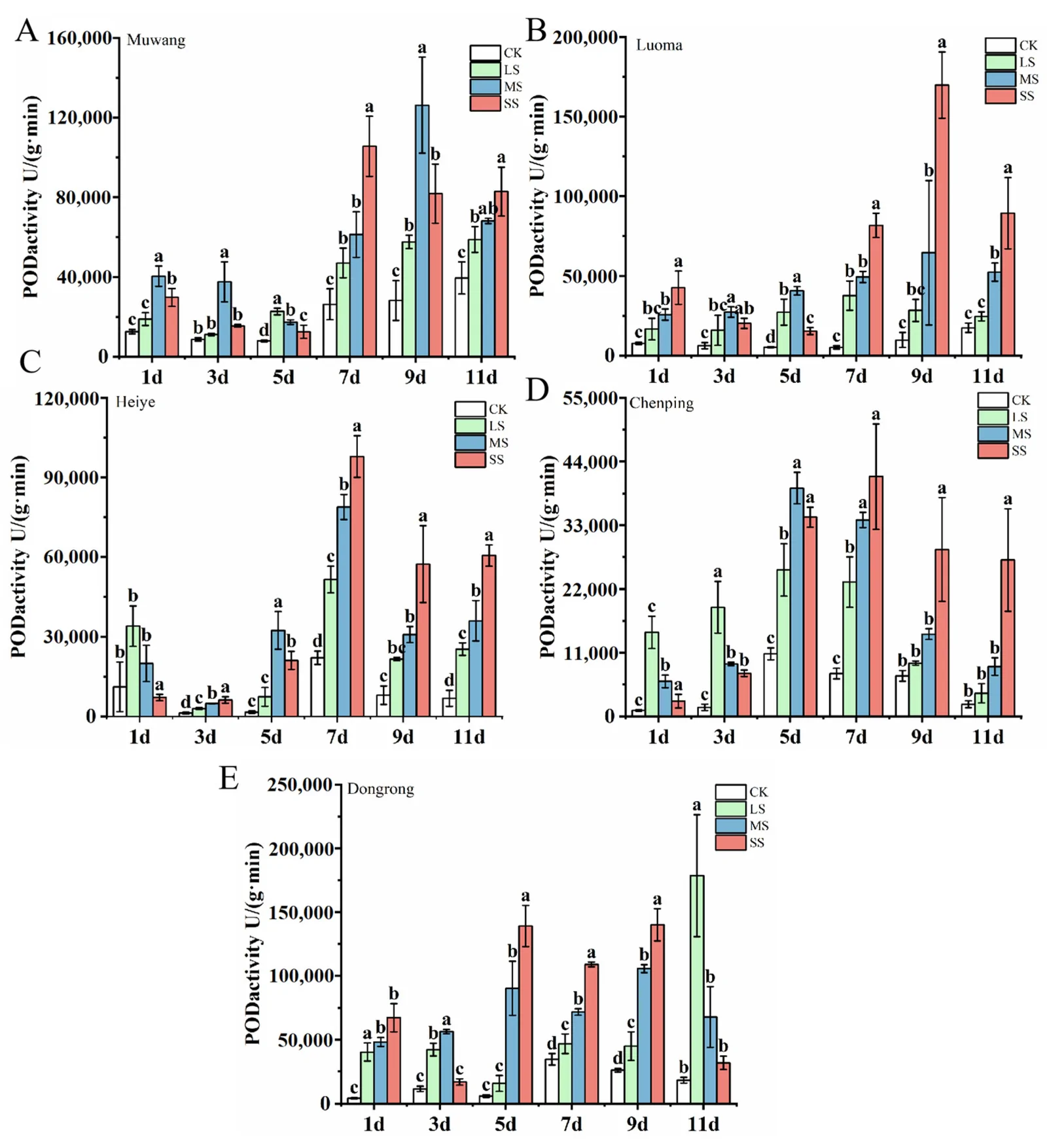
Effect of drought stress on POD activity of five *I. verum* cultivars. (**A**)—Muwang; (**B**)—Louma; (**C**)—Heiye; (**D**)—Chenping; (**E**)—Dongrong. Different lowercase letters indicate significant differences between treatments (*p* < 0.05).

The peroxidase (POD) activities of Muwang, Luoma and Chenping generally increased first and then decreased (Figure 7A,B,D). The POD activity of Heiye showed a trend of decreasing–increasing–decreasing (Figure 7C), while Dongrong’s POD activity presented an overall upward tendency (Figure 7E). On the 11th day of drought stress, POD activities of Muwang under LS, MS and SS treatments were 0.49-, 0.72- and 1.10-fold higher than CK, respectively. The corresponding fold increases were 0.41, 1.99 and 4.11 for Luoma; 2.72, 4.29, and 7.91 for Heiye; 0.90, 3.10 and 11.86 for Chenping; and 8.81, 2.72 and 0.75 for Dongrong.

Dongrong displayed the greatest change in POD activity under LS, indicating that it rapidly activates POD to trigger antioxidant defense systems and mitigate oxidative injury. Its POD activity changed the least under SS, revealing limited enhancement of antioxidant capacity under severe stress. Heiye had the most prominent elevation of POD activity under MS, which suggested this cultivar alleviates membrane lipid peroxidation by boosting POD activity. Chenping exhibited the largest variation in POD activity under SS, reflecting that it requires dramatic upregulation of POD to counteract severe oxidative damage. Luoma showed minimal POD fluctuation under LS, meaning it could maintain oxidative balance without obvious POD upregulation at mild stress. Muwang had the smallest increment of POD activity under moderate stress, with only slight improvement in its antioxidant scavenging capacity.

#### 2.4.3. Effects of Drought Stress on CAT Activity of Five *I. verum* Cultivar Seedlings

**Figure 8:**
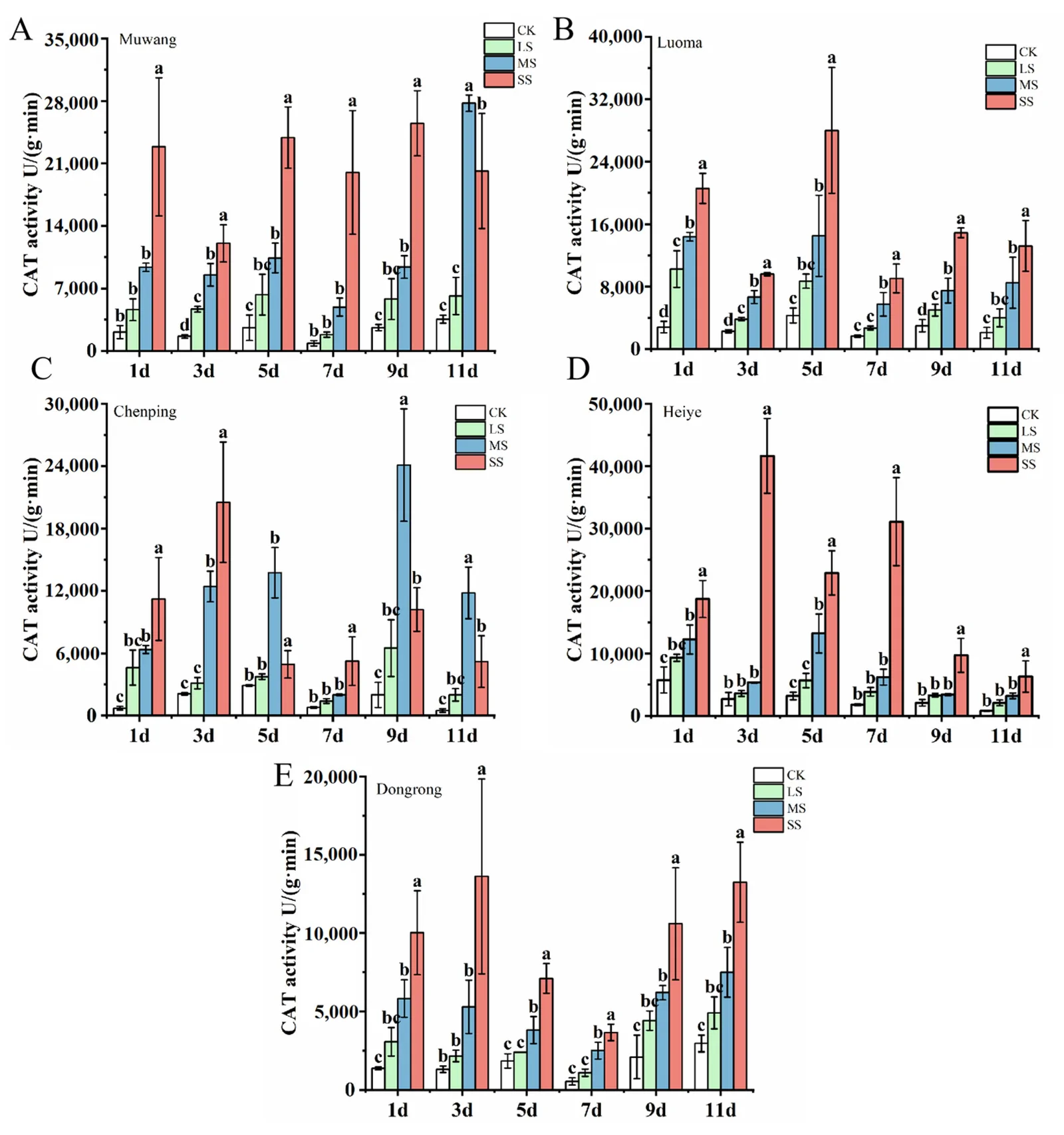
Effects of drought stress on CAT activity of five *I. verum* cultivars. (**A**)—Muwang; (**B**)—Louma; (**C**)—Heiye; (**D**)—Chenping; (**E**)—Dongrong. Different lowercase letters indicate significant differences between treatments (*p* < 0.05).

Catalase (CAT) activity of Muwang remained relatively stable throughout the treatment period (Figure 8A). Luoma’s CAT activity followed a trend of decreasing–increasing–decreasing (Figure 8B). Heiye and Chenping exhibited an initial rise followed by a decline in CAT activity (Figure 8C,D), while Dongrong’s CAT activity changed in the pattern of increasing–decreasing–increasing (Figure 8E). On the 11th day of drought stress, CAT activities of Muwang under LS, MS and SS treatments were 0.73-, 6.82- and 4.68-fold higher than CK, respectively. The corresponding fold increases were 0.90, 3.05 and 5.29 for Luoma; 1.63, 3.00, and 6.88 for Heiye; 3.00, 22.60 and 9.40 for Chenping; and 0.66, 1.54 and 3.49 for Dongrong.

Chenping showed the largest variation range of CAT activity under light, moderate, and severe stress, which indicated that this cultivar generates a robust antioxidant response via marked upregulation of CAT activity to reduce oxidative injury. Dongrong had the slightest fluctuation of CAT activity across all drought treatments, implying it maintains a more stable metabolic state under water deficit and suffers less severe oxidative stress.

### 2.5. Comprehensive Evaluation of Drought Resistance of Five I. verum Cultivar Seedlings

The fuzzy membership function method is an effective method to systematically evaluate the stress resistance of plants by integrating multiple indicators. The larger the average membership function value, the stronger the stress resistance of plants. As shown in Table 1, this study calculated the membership function values of 11 indicators for each variety under different treatments, and then accumulated the membership function values under different treatments to calculate the average value. By comparing the membership function values of each variety, the drought resistance of five *I. verum* cultivar seedlings was comprehensively evaluated. The comprehensive drought resistance of the five *I. verum* cultivar seedlings from high to low was Dongrong > Muwang > Luoma > Heiye > Chenping.

## 3. Discussion

Drought stress affected the growth and yield of *I. verum* seedlings. At present, the effects of drought stress on the physiological and biochemical aspects of *I. verum* are less reported. Therefore, this experiment carried out a study on the physiological response of *I. verum* under drought stress.

Relative water content (RWC) is a vital indicator for evaluating plant water status. Under drought stress, leaf relative water content generally declines, and this parameter can partly mirror the leaf water retention capacity of plants [10]. In the present study, RWC of seedlings from all five *I*. *verum* cultivars declined to different extents after drought treatments, consistent with previous findings on *Blumea balsamifera* (L.) DC. [11] and *Clinacanthus nutans* (Burm.f.) Lindau [12]. Among all tested cultivars, Dongrong exhibited the smallest RWC variation, demonstrating its superior capacity for leaf water retention and water homeostasis regulation. In this study, MDA concentrations under all drought treatments were significantly elevated relative to the control, which aligns with previous reports on *Capparis spinosa* var. *inermis* (DC.) Boiss. [13] and *Mentha piperita* L. [14]. Malondialdehyde (MDA) is a primary byproduct of membrane lipid peroxidation, and its accumulation directly indicates the severity of lipid peroxidation and the extent of cell membrane structural damage [15]. Across the five tested *I*. *verum* cultivars, seedling MDA contents all followed an initial increase followed by a subsequent decline. Heiye exhibited the lowest MDA accumulation, reflecting superior drought tolerance. By contrast, Chenping displayed the largest MDA increments under moderate and severe drought stress, suggesting weaker drought resistance.

Proline, soluble proteins, and soluble sugars serve as major plant osmoprotectants that preserve cell turgor and support normal cell growth [16]. In this experiment, the proline content in the leaves of five *I. verum* cultivars increased gradually with the aggravation of drought stress. Under water stress, proline concentrations in *Illicium difengpi* increased to improve drought tolerance, a trend consistent with the findings of the present study [17]. Soluble protein contents exhibited an initial rise followed by a decline, which matched the variation pattern observed in *Bupleurum chinense* [18]. Soluble sugars can lower cellular water potential and improve plants’ water absorption and retention capacity, thereby boosting stress tolerance. Under drought stress, soluble sugar concentrations in *Taxus yunnanensis* continuously accumulated as water stress intensified [19], a trend consistent with the findings of the present study. Luoma seedlings accumulated the highest levels of osmotic adjustment substances, suggesting they synthesize abundant osmolytes under drought stress to adjust cellular osmotic potential, sustain cellular water balance, and preserve structural integrity. By contrast, Chenping seedlings exhibited the lowest accumulation of osmotic regulators and failed to effectively mitigate drought-induced injury via osmotic adjustment, indicating its relatively weak drought tolerance.

Under drought stress, plants can alleviate oxidative damage and preserve the integrity of cellular structures by boosting the activities of antioxidant protective enzymes, including superoxide dismutase (SOD), peroxidase (POD), and catalase (CAT) [20].Under drought stress, the activities of SOD, POD, and CAT in *Dendrobium sinenses* increased significantly [21]. Antioxidant enzyme activities were elevated to coordinate intracellular oxidative metabolism, sustain metabolic homeostasis, and support normal plant growth. The activities of the three antioxidant enzymes in seedlings of all five *I. verum* cultivars increased relative to the control, demonstrating that every variety adapts to drought by upregulating protective enzyme activities. SOD activity rose steadily in Luoma and Chenping seedlings, whereas Muwang, Heiye, and Dongrong exhibited an initial increase followed by a decline. This indicates the antioxidant defense system of all five *I. verum* seedlings was fully activated, and cellular oxidative damage was alleviated via elevated SOD activity. Li et al. (2022) [22] showed that T-SOD, POD, and CAT activities in *Platycodon grandiflorum* were markedly higher under drought conditions than in the control group throughout the treatment period. In this study, POD activity in all five *I. verum* seedling cultivars except Dongrong presented an initial increase followed by a decline, revealing that the plant antioxidant system exhibits stage-specific responses to drought stress. The shift of POD activity from rising to falling signals suppressed function of the antioxidant enzyme system and weakened antioxidant capacity, consistent with previous findings on *Chrysanthemum morifoliu* Ramat [23]. Catalase (CAT) acts as a vital antioxidant protective enzyme in plants to mitigate oxidative injury and sustain intracellular redox homeostasis. [24]. In this study, Chenping exhibited the highest increase in CAT activity under all drought stress gradients. It alleviates drought-induced oxidative injury by drastically enhancing CAT-mediated antioxidant capacity, representing a typical stress-responsive drought resistance strategy. In contrast, Dongrong showed the slightest fluctuation in CAT activity across all drought treatments, maintaining more stable metabolism and antioxidant status with less oxidative stress damage, which confers stronger persistent drought tolerance. Muwang exhibited generally stable CAT activity, consistent with the variation trend observed in the alfalfa cultivar Dimitra (*Medicago sativa* L.) [25]. As drought stress persisted, the activities of the three enzymes declined in the late treatment stage, consistent with observations in *Malania oleifera* [26]. This demonstrates that prolonged late-stage drought impairs the seedling antioxidant enzyme system and inflicts irreversible harm on *I. verum* seedlings.

It is usually considered unreliable to evaluate the drought resistance of plants only by a single index [27]. Therefore, the fuzzy membership function method was used in this experiment to systematically evaluate the stress resistance of five cultivars of *I. verum*. The greater the average membership function value, the stronger the resistance of the plant [28]. Zhang et al. (2021) [29] assessed drought tolerance across ten *Iris germanica* cultivars using subordinate function analysis, and their results proved that the cultivar ‘Little Dream’ possessed superior drought resistance compared with the rest of the tested materials. By comparing the membership function values of each variety, the drought resistance of five *I. verum* cultivars was comprehensively evaluated, and the comprehensive drought resistance of five *I. verum* cultivars was obtained. In this experiment, Dongrong seedlings had the strongest drought resistance.

## 4. Materials and Methods

### 4.1. Research Overview

The experiment was carried out in the scientific research base of the research team of traditional Chinese medicine resources of Guangxi University of traditional Chinese medicine from July to August 2025 (108°50′ E, 22°81′ N). A controlled greenhouse was set up in the base, with a temperature of (25 ± 2) °C and a humidity of (60 ± 5)%.

The experimental material was derived from the Gulong Chunyu *Illicium verum* Hook.f. planting professional cooperative in Gulong Town, Tengxian County. It was identified as one-year-old seedlings of *Illicium verum* Hook.f. by Huang Ding researcher of Guangxi University of Traditional Chinese Medicine. The five *I. verum* cultivars used in the experiment were Muwang, Luoma, Heiye, Chenping, and Dongrong.

### 4.2. Drought Stress Treatment

One-year-old seedlings of five well-growing *I. verum* cultivars were selected. The seedling roots were rinsed with distilled water, and the seedlings were transferred to plastic pots filled with Hoagland nutrient solution. Four PEG-6000 concentration levels were set: 0%, 3%, 6% and 9%, corresponding to control (CK), light drought stress (LS), moderate drought stress (MS) and severe drought stress (SS), respectively. Five seedlings were arranged for each drought treatment.

Sampling was conducted at 1, 3, 5, 7, 9 and 11 days after PEG-6000 treatment. Three plants were randomly selected at each sampling time point. Their leaves were cut into pieces, snap-frozen in liquid nitrogen, and then stored in a −80 °C refrigerator for subsequent determination of all physiological indices. Each treatment was measured in three biological replicates.

### 4.3. Item Determination and Methods

#### 4.3.1. Determination of Leaf Relative Water Content

The relative water content (RWC) of leaves was determined by the weighing method. Three replicates of fresh leaf samples were collected and weighed to record fresh weight (FW). The leaves were immersed in pure water for 24 h before measuring turgid weight (TW). Subsequently, the leaf samples were oven-dried at 80 °C to constant weight and weighed to obtain dry weight (DW). Relative water content was calculated according to the following formula: RWC (%) = (FW − DW)/(TW − DW) × 100% [30].

#### 4.3.2. Determination of Malondialdehyde Content

MDA content was measured by spectrophotometry. Briefly, 0.05 g leaf tissue was ground with 1 mL of 5% trichloroacetic acid (TCA). The homogenate was centrifuged at 3000 r/min for 10 min. Then 0.5 mL supernatant was transferred into a test tube and mixed with 0.75 mL of 0.67% thiobarbituric acid (TBA). The mixture was heated in a boiling water bath at 100 °C for 30 min, cooled to room temperature and centrifuged again to obtain the test supernatant. Distilled water was used as the blank control. The absorbance values of the supernatant were detected at 450 nm, 532 nm, and 600 nm, and the MDA concentration was calculated according to the corresponding formula [31].

#### 4.3.3. Determination of Proline Content

Proline content was determined using the acid ninhydrin method. Briefly, 0.05 g leaf samples was weighed and mixed with 1 mL of 3% sulfosalicylic acid solution, followed by extraction in a boiling water bath for 10 min. After cooling, the mixture was filtered to obtain proline extract.

Subsequently, 0.5 mL of the extract was transferred to a new test tube, mixed with 0.5 mL glacial acetic acid and 0.5 mL acid ninhydrin reagent, and heated in a boiling water bath for 30 min. Once cooled, 1 mL toluene was added, and the tube was shaken vigorously for 30 s before standing still for a short while. The upper organic phase was collected into a centrifuge tube and centrifuged at 3000 r/min for 5 min. Toluene was used as the blank control. The absorbance was measured at 520 nm with a spectrophotometer (Ultraviolet spectrophotometer, Model 7521812034, Shanghai Jinghua Scientific Instrument Co., Ltd., Shanghai, China), and proline concentration was quantified based on the standard curve [32].

#### 4.3.4. Determination of Soluble Protein Content

Soluble protein content was determined by the Coomassie Brilliant Blue G-250 method (Bradford method). Briefly, 0.05 g fresh leaf tissue was ground with 1.6 mL pre-cooled 0.05 mol/L phosphate buffer saline (PBS, pH 7.8) under freezing conditions. The homogenate was transferred into a 2 mL centrifuge tube and centrifuged at 12,000 r/min for 10 min at 4 °C; the collected supernatant served as protein extract.

Subsequently, 0.1 mL of the extract was mixed with 5 mL Coomassie Brilliant Blue G-250 reagent. After standing for 2 min, absorbance was measured at 595 nm. Soluble protein concentration was quantified based on the standard curve [33].

#### 4.3.5. Determination of Soluble Sugar Content

Soluble sugar content was determined by the anthrone colorimetric method. Preparation of anthrone reagent: Mix 84 mL concentrated sulfuric acid with 16 mL distilled water, then add 0.15 g anthrone, and store the reagent away from light.

Extraction of soluble sugar: 0.1 g fresh leaf tissue was weighed and mixed with 5 mL distilled water, followed by two rounds of extraction in a boiling water bath for 30 min each. After cooling, the mixture was centrifuged. The supernatant was transferred into a 50 mL centrifuge tube, and the volume was made up to the mark with hot distilled water.

Color reaction: 0.1 mL extract was mixed thoroughly with 3 mL anthrone reagent, incubated in a boiling water bath for 10 min, and cooled down. The absorbance was measured at 620 nm, and soluble sugar concentration was quantified using the standard curve [31].

#### 4.3.6. Determination of Superoxide Dismutase Activity

SOD activity was assayed using the nitro blue tetrazolium (NBT) method.

(1) Enzyme extraction: a total of 0.05 g fresh leaf tissue was placed into a pre-cooled mortar, mixed with 1.6 mL pre-cooled 0.05 mol/L PBS (pH 7.8), and ground into homogenate on an ice bath. The homogenate was transferred to a 2 mL centrifuge tube and centrifuged at 12,000 r/min for 20 min at 4 °C; the resulting supernatant was the crude enzyme extract.

(2) Color reaction system: the reaction mixture contained 1.5 mL of 0.05 mol/L PBS (pH 7.8), 0.3 mL of 130 mM methionine solution, 0.3 mL of 750 μmol/L NBT solution, 0.3 mL of 100 μmol/L EDTA-Na_2_, 0.3 mL of 20 μmol/L riboflavin, 0.05 mL enzyme extract, and 0.25 mL distilled water. After thorough mixing, one control tube was kept in darkness, while the remaining tubes were exposed to 4,000 lx fluorescent light (General Environmental Laboratory (Plant Growth Chamber), Model MLR-35211-PC, Panasonic Healthcare Co., Ltd., Toon, Ehime, Japan) for 20 min to initiate the photoreaction [34].

(3) Measurement and calculation of SOD activity: after the reaction terminated, absorbance was detected at 560 nm using the dark-treated tube as the blank control. One unit (U) of SOD activity was defined as the amount of enzyme inhibiting the photoreduction of NBT by 50%. SOD activity was calculated according to the corresponding formula.

#### 4.3.7. Determination of Peroxidase (POD) Activity

POD activity was determined via the guaiacol colorimetric method.

(1) Enzyme extraction: the enzyme extraction procedure was consistent with that described in Section 4.3.7.

(2) Preparation and measurement of reaction system: first, 56 μL guaiacol was dissolved in 1 mL of 95% ethanol, followed by the addition of 100 mL 0.2 mol/L PBS (pH 6.0) and 38 μL 30% H_2_O_2_ to prepare the POD reaction solution. A total of 200 μL reaction solution was mixed with 10 μL crude enzyme extract, and the absorbance change at 470 nm within 1 min was recorded. One unit (U) of POD activity was defined as a 0.01 increase in absorbance per minute [35].

#### 4.3.8. Determination of Catalase (CAT) Activity

(1) Enzyme extraction: the enzyme extraction method was identical to that described in Section 4.3.7.

(2) Prepare CAT reaction mixture by mixing 0.1546 mL of 30% H_2_O_2_ with 100 mL of 0.15 mol/L PBS (pH 7.0). Then 200 μL reaction mixture was combined with 10 μL enzyme extract, and the absorbance change at 240 nm within 1 min was recorded. One unit (U) of CAT activity was defined as a 0.01 decrease in absorbance per minute. Catalase activity was expressed as U/g FW [36].

### 4.4. Membership Function Method

The analysis of the fuzzy mathematics membership function method was based on the experimental results on the 11th day of drought stress. The values of each index were converted into membership function or inverse membership function values for drought resistance comparison. The drought resistance membership function values of each index were accumulated, and the average value was obtained. The greater the average value, the stronger the drought resistance. If the index is positively correlated with drought resistance, it is calculated according to Formula (1); if the index is negatively correlated with drought resistance, it is calculated according to Formula (2). The calculation formula is as follows [37]:

X1 = (X − X min)/(X max − X min)(1)

X2 = 1 − (X − X min)/(X max − X min)(2)

In the formula, X1 and X2 are the membership function values, X is the measured value/control value of a certain index, X min is the minimum value in the test material X, and X max is the maximum value in the test material X.

### 4.5. Statistical Analysis

Excel 2020 was used to process the data, and SPSS 27.0 was used for analysis of variance and Duncan multiple comparison to test the significant differences between different treatments (*p* < 0.05). Drawing with OrigrinPro 2024.

## 5. Conclusions

In this experiment, the relative water content of seedlings of the five *I. verum* cultivars showed a decreasing trend, and long-term drought severely disrupted plant water balance. Malondialdehyde content increased continuously, aggravating membrane lipid peroxidation. Soluble protein, soluble sugar, and proline accumulated in large quantities; through synergistic effects, the three substances maintained cellular osmotic balance and alleviated drought-induced damage to cell membranes. The activities of antioxidant enzymes SOD, POD and CAT in leaves of seedlings from different *I. verum* cultivars increased gradually. These enzymes synergistically scavenge intracellular free radicals and alleviate the damage of free radicals to cell membrane systems. Combined with the eight indexes determined by the seedlings of five *I. verum* cultivars, the comprehensive evaluation of drought resistance was made, and the comprehensive ranking was Dongrong > Muwang > Luoma > Heiye > Chenping. In the comprehensive analysis, the drought resistance of Dongrong seedlings was the strongest, and the drought resistance of Chenping seedlings was the weakest.

## Figures and Tables

**Table 1:** Subordinate function and comprehensive evaluation results of drought resistance index of five *I. verum* cultivar seedlings.

Variety	Muwang	Luoma	Heiye	Chenping	Dongrong
RWC	0.843	0.057	0.833	0.055	0.905
MDA	0.889	0.366	1.000	0.060	0.525
Pro	0.668	0.724	0.299	0.000	0.320
Sp	0.381	0.537	0.017	0.194	0.947
Ss	0.404	0.855	0.214	0.106	0.590
SOD	0.687	0.744	0.000	0.797	0.654
POD	0.014	0.219	0.640	0.575	0.520
CAT	0.160	0.160	0.351	1.000	0.000
overall merit	0.506	0.458	0.419	0.348	0.558
sort	2	3	4	5	1

## Data Availability

The original contributions presented in this study are included in the article. Further inquiries can be directed to the corresponding author.
